# Genome Sequence of Escherichia coli KI683, Isolated from a Urosepsis Patient

**DOI:** 10.1128/MRA.01297-19

**Published:** 2020-02-27

**Authors:** Nathalie Stefani, Volker Schroeckh, Ute Neugebauer, Jürgen Bohnert, Axel A. Brakhage

**Affiliations:** a Leibniz Institute for Natural Product Research and Infection Biology–Hans Knöll Institute, Molecular and Applied Microbiology, Jena, Germany; b Institute for Microbiology, Friedrich Schiller University, Jena, Germany; c Center for Sepsis Control and Care, Jena University Hospital, Jena, Germany; d Institute of Medical Microbiology, Jena University Hospital, Jena, Germany; e Friedrich Loeffler Institute of Medical Microbiology, Greifswald University Hospital, Greifswald, Germany; University of Arizona

## Abstract

Escherichia coli KI683 was isolated from blood of a patient who developed septicemia as a complication of a urinary tract infection. Genome sequencing resulted in three contigs with a total genome size of 5,243,173 bp encoding 5,143 genes.

## ANNOUNCEMENT

Escherichia coli strains colonizing implants in a given patient mostly derive from E. coli infections of surgical wounds, the respiratory system, and the urinary tract (1). The bacteria are spread via the bloodstream to susceptible spaces in soft and hard tissues where they form difficult-to-treat biofilms (2). Therefore, E. coli strains found on implant material are highly similar to those isolated from patients with sepsis or urinary tract infection (3). Since E. coli strain KI683 was isolated from a sepsis patient, it represents an ideal candidate for identifying adhesion mechanisms of E. coli to abiotic surfaces by genome comparison to other strains.

E. coli KI683 was isolated at the Jena University Hospital in December 2014 from blood of a male patient diagnosed with sepsis. Isolation, identification, and cultivation conditions were published elsewhere (4). Briefly, positive blood cultures were determined with the Bactec FX instrument (BD Diagnostics, Heidelberg, Germany), and identification of the E. coli isolate was achieved using the Vitek 2 system (bioMérieux, Nürtingen, Germany).

E. coli KI683 cells were stored at −80°C in a cryobank vial (Mast Diagnotics GmbH, Reinfeld, Germany). One bead of this vial was added to LB medium, and the bacteria sticking to this bead were cultured at 37°C with shaking for 16 h. Five aliquots of 300 μl were removed from the culture; the genomic DNA (gDNA) was extracted using the MagAttract high-molecular-weight (HMW) DNA kit (Qiagen, Hilden, Germany) and then pooled for further processing. Generation of a genomic library, single-molecule real-time (SMRT) sequencing, and annotation of the genome were performed by Genewiz (South Plainfield, NJ). Briefly, sheared gDNA was used to generate a SMRTbell library with an average size of 10 kb per DNA fragment. Genome sequencing was performed in one SMRT cell on a PacBio Sequel instrument (Pacific Biosciences, Menlo Park, CA), yielding 747,843 polymer reads in total with an average read length of 12,931 bp. Raw data were quality controlled and *de novo* assembled using Canu v1.7 (https://github.com/marbl/canu) (5).

Further polishing of the contigs was done with raw subreads by Arrow 2.2.2 (Pacific Biosciences). Polished contigs were annotated using Prokka v1.13 (https://github.com/tseemann/prokka) (6). Default parameters were used for all bioinformatics software.

The derived genomic sequence of E. coli KI683 was distributed on three contigs, with sizes of 5,110,765, 114,238 (circularized), and 18,712 bp, respectively (1,457× coverage; *N*_50_, 5,110,765 bp). This resulted in a total genome size of 5,243,173 bp, with a G+C content of 51%. The chromosome encodes 5,143 genes, consisting of 4,834 coding DNA sequences (CDSs), 91 tRNAs, 217 miscellaneous RNAs, and 1 transfer-messenger RNA (tmRNA).

### Data availability.

The sequence data were deposited under DDBJ/ENA/GenBank under the study number PRJEB34704. The raw sequence reads are available under the accession number ERX3577898.
